# Epidemiology, ventilation management and outcomes of COVID–19 ARDS patients versus patients with ARDS due to pneumonia in the Pre–COVID era

**DOI:** 10.1186/s12931-024-02910-2

**Published:** 2024-08-17

**Authors:** Fleur–Stefanie L. I. M. van der Ven, Siebe G. Blok, Luciano C. Azevedo, Giacomo Bellani, Michela Botta, Elisa Estenssoro, Eddy Fan, Juliana Carvalho Ferreira, John G. Laffey, Ignacio Martin–Loeches, Ana Motos, Tai Pham, Oscar Peñuelas, Antonio Pesenti, Luigi Pisani, Ary Serpa Neto, Marcus J. Schultz, Antoni Torres, Anissa M. Tsonas, Frederique Paulus, David M. P. van Meenen, and for the ERICC–, Amadeu Martinez, Livia Leal, Antonio Jorge Pereira, Marcelo de Oliveira Maia, Josè Aires Neto, Claudio Piras, Eliana Bernadete Caser, Cora Lavigne Moreira, Pablo Braga Gusman, Dyanne Moysés Dalcomune, Alexandre Guilherme Ribeiro de Carvalho, Louise Aline Romão Gondim, Lívia Mariane Castelo Branco Reis, Daniel da Cunha Ribeiro, Leonardo de Assis Simões, Rafaela Siqueira Campos, José Carlos Fernandez Versiani dos Anjos, Frederico Bruzzi Carvalho, Rossine Ambrosio Alves, Lilian Batista Nunes, Álvaro Réa-Neto, Mirella Cristine de Oliveira, Luana Tannous, Brenno Cardoso Gomes, Fernando Borges Rodriguez, Priscila Abelha, Marcelo E Lugarinho, Andre Japiassu, Hélder Konrad de Melo, Elton Afonso Lopes, Pedro Varaschin, Vicente Cés de Souza Dantas, Marcos Freitas Knibel, Micheli Ponte, Pedro Mendes de Azambuja Rodrigues, Rubens Carmo Costa Filho, Felipe Saddy, Théia Forny Wanderley Castellões, Suzana Alves Silva, Luiz Antonio Gomes Osorio, Dora Mannarino, Rodolfo Espinoza, Cassia Righy, Marcio Soares, Jorge Salluh, Lilian Tanaka, Daniel Aragão, Maria Eduarda Tavares, Maura Goncalves Pereira Kehdi, Valéria Maria Campos Rezende, Roberto Carlos Cruz Carbonell, Cassiano Teixeira, Roselaine Pinheiro de Oliveira, Juçara Gasparetto Maccari, Priscylla Souza Castro, Paula Berto, Patricia Schwarz, André Peretti Torelly, Thiago Lisboa, Edison Moraes, Felipe Dal-Pizzol, Cristiane Tomasi Damiani, Cristiane Ritter, Juliana Carvalho Ferreira, Ramon Teixeira Costa, Pedro Caruso, Cristina Prata Amendola, Amanda Maria RR de Oliveira, Ulysses VA Silva, Luciana Coelho Sanches, Rosana DS Almeida, Luciano Cesar Azevedo, Marcelo Park, Guilherme Schettino, Murillo Santucci Assunção, Eliezer Silva, Carlos Eduardo Barboza, Antonio Paulo Nassar Junior, Paulo Fernando GMMarzocchi Tierno, Luis Marcelo Malbouisson, Lucas Oliveira, Davi Cristovao, Manoel Leitão Neto, Ênio Rego, Fernanda Eugênia Fernandes, Marcelo Luz Pereira Romano, Alexandre Biasi Cavalcanti, Dalton de Souza Barros, Érica Aranha Suzumura, Karla Loureiro Meira, Gustavo Affonso de Oliveira, Paula Menezes Luciano, Evelin Drociunas Pacheco, Bruno Franco Mazza, Flavia Ribeiro Machado, Elaine Ferreira, Ronaldo Batista dos Santos, Alexandra Siqueira Colombo, Antonio Carlos Nogueira, Juliana Baroni Fernandes, Raquel Siqueira Nóbrega, Barbara do CS Martins, Francisco Soriano, Rafaela Deczka Morsch, Andre Luiz Baptiston Nunes, Juliano Pinheiro de Almeida, Ludhmila Hajjar, Sílvia Moulin, Fábio Poianas Giannini, Andre Luiz Baptiston Nunes, LUNG SAFE–, Fernando Rios, Frank Van Haren, T Sottiaux, Fredy S Lora, Luciano C Azevedo, P Depuydt, Eddy Fan, Guillermo Bugedo, Haibo Qiu, Marcos Gonzalez, Juan Silesky, Vladimir Cerny, Jonas Nielsen, Manuel Jibaja, Tài Pham, Hermann Wrigge, Dimitrios Matamis, Jorge Luis Ranero, SM Hashemian, Pravin Amin, Kevin Clarkson, Giacomo Bellani, Kiyoyasu Kurahashi, Asisclo Villagomez, Amine Ali Zeggwagh, Leo M Heunks, Jon Henrik Laake, Jose Emmanuel Palo, Antero do Vale Fernandes, Dorel Sandesc, Yaasen Arabi, Vesna Bumbasierevic, Jose A Lorente, Anders Larsson, Lise Piquilloud, Fekri Abroug, Daniel F McAuley, Lia McNamee, Javier Hurtado, Ed Bajwa, Gabriel Démpaire, Guy M Francois, Hektor Sula, Lordian Nunci, Alma Cani, Alan Zazu, Christian Dellera, Carolina S Insaurralde, Risso V Alejandro, Julio Daldin, Mauricio Vinzio, Ruben O Fernandez, Luis P Cardonnet, Lisandro R Bettini, Mariano Carboni Bisso, Emilio M Osman, Mariano G Setten, Pablo Lovazzano, Javier Alvarez, Veronica Villar, Cesar Milstein, Norberto C Pozo, Nicolas Grubissich, Gustavo A Plotnikow, Daniela N Vasquez, Santiago Ilutovich, Norberto Tiribelli, Ariel Chena, Carlos A Pellegrini, María G Saenz, Elisa Estenssoro, Matias Brizuela, Hernan Gianinetto, Pablo E Gomez, Valeria I Cerrato, Marco G Bezzi, Silvina A Borello, Flavia A Loiacono, Adriana M Fernandez, Serena Knowles, Claire Reynolds, Deborah M Inskip, Jennene J Miller, Jing Kong, Christina Whitehead, Shailesh Bihari, Aylin Seven, Amanda Krstevski, Helen J Rodgers, Rebecca T Millar, Toni E Mckenna, Irene M Bailey, Gabrielle C Hanlon, Anders Aneman, Joan M Lynch, Raman Azad, John Neal, Paul W Woods, Brigit L Roberts, Mark R Kol, Helen S Wong, Katharina C Riss, Thomas Staudinger, Xavier Wittebole, Caroline Berghe, Pierre A Bulpa, Alain M Dive, Rik Verstraete, Herve Lebbinck, Pieter Depuydt, Joris Vermassen, Philippe Meersseman, Helga Ceunen, Jonas I Rosa, Daniel O Beraldo, Claudio Piras, Adenilton MR Ampinelli, Antonio P Nassar, Sergio Mataloun, Marcelo Moock, Marlus M Thompson, Claudio H Gonçalves, Ana Carolina P Antônio, Aline Ascoli, Rodrigo S Biondi, Danielle C Fontenele, Danielle Nobrega, Vanessa M Sales, Suresh Shindhe, Dk Maizatul Aiman BPg Hj Ismail, John Laffey, Francois Beloncle, Kyle G Davies, Rob Cirone, Venika Manoharan, Mehvish Ismail, Ewan C Goligher, Mandeep Jassal, Erin Nishikawa, Areej Javeed, Gerard Curley, Nuttapol Rittayamai, Matteo Parotto, Niall D Ferguson, Sangeeta Mehta, Jenny Knoll, Antoine Pronovost, Sergio Canestrini, Alejandro R Bruhn, Patricio H Garcia, Felipe A Aliaga, Pamela A Farías, Jacob S Yumha, Claudia A Ortiz, Javier E Salas, Alejandro A Saez, Luis D Vega, Eduardo F Labarca, Felipe T Martinez, Nicolás G Carreño, Pilar Lora, Haitao Liu, Haibo Qiu, Ling Liu, Rui Tang, Xiaoming Luo, Youzhong An, Huiying Zhao, Yan Gao, Zhe Zhai, Zheng L Ye, Wei Wang, Wenwen Li, Qingdong Li, Ruiqiang Zheng, Wenkui Yu, Juanhong Shen, Xinyu Li, Tao Yu, Weihua Lu, Ya Q Wu, Xiao B Huang, Zhenyang He, Yuanhua Lu, Hui Han, Fan Zhang, Renhua Sun, Hua X Wang, Shu H Qin, Bao H Zhu, Jun Zhao, Jian Liu, Bin Li, Jing L Liu, Fa C Zhou, Qiong J Li, Xing Y Zhang, Zhou Li-Xin, Qiang Xin-Hua, Liangyan Jiang, Yuan N Gao, Xian Y Zhao, Yuan Y Li, Xiao L Li, Chunting Wang, Qingchun Yao, Rongguo Yu, Kai Chen, Huanzhang Shao, Bingyu Qin, Qing Q Huang, Wei H Zhu, Ai Y Hang, Ma X Hua, Yimin Li, Yonghao Xu, Yu D Di, Long L Ling, Tie H Qin, Shou H Wang, Junping Qin, Yi Han, Suming Zhou, Monica P Vargas, Juan I Silesky Jimenez, Manuel A González Rojas, Jaime E Solis-Quesada, Christian M Ramirez-Alfaro, Jan Máca, Peter Sklienka, Jakob Gjedsted, Aage Christiansen, Jonas Nielsen, Boris G Villamagua, Miguel Llano, Philippe Burtin, Gautier Buzancais, Pascal Beuret, Nicolas Pelletier, Satar Mortaza, Alain Mercat, Jonathan Chelly, Sébastien Jochmans, Nicolas Terzi, Cédric Daubin, Guillaume Carteaux, Nicolas de Prost, Jean-Daniel Chiche, Fabrice Daviaud, Tai Pham, Muriel Fartoukh, Guillaume Barberet, Jerome Biehler, Jean Dellamonica, Denis Doyen, Jean-Michel Arnal, Anais Briquet, Sami Hraiech, Laurent Papazian, Arnaud Follin, Damien Roux, Jonathan Messika, Evangelos Kalaitzis, Laurence Dangers, Alain Combes, Siu-Ming Au, Gaetan Béduneau, Dorothée Carpentier, Elie H Zogheib, Herve Dupont, Sylvie Ricome, Francesco L Santoli, Sebastien L Besset, Philippe Michel, Bruno Gelée, Pierre-Eric Danin, Bernard Goubaux, Philippe J Crova, Nga T Phan, Frantz Berkelmans, Julio C Badie, Romain Tapponnier, Josette Gally, Samy Khebbeb, Jean-Etienne Herbrecht, Francis Schneider, Pierre-Louis M Declercq, Jean-Philippe Rigaud, Jacques Duranteau, Anatole Harrois, Russell Chabanne, Julien Marin, Charlene Bigot, Sandrine Thibault, Mohammed Ghazi, Messabi Boukhazna, Salem Ould Zein, Jack R Richecoeur, Daniele M Combaux, Fabien Grelon, Charlene Le Moal, Elise P Sauvadet, Adrien Robine, Virginie Lemiale, Danielle Reuter, Martin Dres, Alexandre Demoule, Dany Goldgran-Toledano, Loredana Baboi, Claude Guérin, Ralph Lohner, Jens Kraßler, Susanne Schäfer, Kai D Zacharowski, Patrick Meybohm, Andreas W Reske, Philipp Simon, Hans-Bernd F Hopf, Michael Schuetz, Thomas Baltus, Metaxia N Papanikolaou, Theonymfi G Papavasilopoulou, Giannis A Zacharas, Vasilis Ourailogloy, Eleni K Mouloudi, Eleni V Massa, Eva O Nagy, Electra E Stamou, Ellada V Kiourtzieva, Marina A Oikonomou, Luis E Avila, Cesar A Cortez, Johanna E Citalán, Sameer A Jog, Safal D Sable, Bhagyesh Shah, Mohan Gurjar, Arvind K Baronia, Mohammedfaruk Memon, Radhakrishnan Muthuchellappan, Venkatapura J Ramesh, Anitha Shenoy, Ramesh Unnikrishnan, Subhal B Dixit, Rachana V Rhayakar, Nagarajan Ramakrishnan, Vallish K Bhardwaj, Heera L Mahto, Sudha V Sagar, Vijayanand Palaniswamy, Deeban Ganesan, Seyed Mohammadreza Hashemian, Hamidreza Jamaati, Farshad Heidari, Edel A Meaney, Alistair Nichol, Karl M Knapman, Donall O’Croinin, Eimhin S Dunne, Dorothy M Breen, Kevin P Clarkson, Rola F Jaafar, Rory Dwyer, Fahd Amir, Olaitan O Ajetunmobi, Aogan C O’Muircheartaigh, Colin S Black, Nuala Treanor, Daniel V Collins, Wahid Altaf, Gianluca Zani, Maurizio Fusari, Savino Spadaro, Carlo A Volta, Romano Graziani, Barbara Brunettini, Salvatore Palmese, Paolo Formenti, Michele Umbrello, Andrea Lombardo, Elisabetta Pecci, Marco Botteri, Monica Savioli, Alessandro Protti, Alessia Mattei, Lorenzo Schiavoni, Andrea Tinnirello, Manuel Todeschini, Antonino Giarratano, Andrea Cortegiani, Sara Sher, Anna Rossi, Massimo M Antonelli, Luca M Montini, Paolo Casalena, Sergio Scafetti, Giovanna Panarello, Giovanna Occhipinti, Nicolò Patroniti, Matteo Pozzi, Roberto R Biscione, Michela M Poli, Ferdinando Raimondi, Daniela Albiero, Giulia Crapelli, Eduardo Beck, Vincenzo Pota, Vincenzo Schiavone, Alexandre Molin, Fabio Tarantino, Giacomo Monti, Elena Frati, Lucia Mirabella, Gilda Cinnella, Tommaso Fossali, Riccardo Colombo, Pierpaolo Terragni, Ilaria Pattarino, Francesco Mojoli, Antonio Braschi, Erika E Borotto, Andrea N Cracchiolo, Daniela M Palma, Francesco Raponi, Giuseppe Foti, Ettore R Vascotto, Andrea Coppadoro, Luca Brazzi, Leda Floris, Giorgio A Iotti, Aaron Venti, Osamu Yamaguchi, Shunsuke Takagi, Hiroki N Maeyama, Eizo Watanabe, Yoshihiro Yamaji, Kazuyoshi Shimizu, Kyoko Shiozaki, Satoru Futami, Sekine Ryosuke, Koji Saito, Yoshinobu Kameyama, Keiko Ueno, Masayo Izawa, Nao Okuda, Hiroyuki Suzuki, Tomofumi Harasawa, Michitaka Nasu, Tadaaki Takada, Fumihito Ito, Shin Nunomiya, Kansuke Koyama, Toshikazu Abe, Kohkichi Andoh, Kohei Kusumoto, Akira Hirata, Akihiro Takaba, Hiroyasu Kimura, Shuhei Matsumoto, Ushio Higashijima, Hiroyuki Honda, Nobumasa Aoki, Hiroshi Imai, Yasuaki Ogino, Ichiko Mizuguchi, Kazuya Ichikado, Kenichi Nitta, Katsunori Mochizuki, Tomoaki Hashida, Hiroyuki Tanaka, Tomoyuki Nakamura, Daisuke Niimi, Takeshi Ueda, Yozo Kashiwa, Akinori Uchiyama, Olegs Sabelnikovs, Peteris Oss, Youssef Haddad, Kong Y Liew, Silvio A Ñamendys-Silva, Yves D Jarquin-Badiola, Luis A Sanchez-Hurtado, Saira S Gomez-Flores, Maria C Marin, Asisclo J Villagomez, Jordana S Lemus, Jonathan M Fierro, Mavy Ramirez Cervantes, Francisco Javier Flores Mejia, Daniel R Gonzalez, Dulce M Dector, Claudia R Estrella, Jorge R Sanchez-Medina, Alvaro Ramirez-Gutierrez, Fernando G George, Janet S Aguirre, Juan A Buensuseso, Manuel Poblano, Tarek Dendane, Amine Ali Zeggwagh, Hicham Balkhi, Mina Elkhayari, Nacer Samkaoui, Hanane Ezzouine, Abdellatif Benslama, Mourad Amor, Wajdi Maazouzi, Nedim Cimic, Oliver Beck, Monique M Bruns, Jeroen A Schouten, Myra Rinia, Monique Raaijmakers, Leo M Heunks, Hellen M Van Wezel, Serge J Heines, Marc P Buise, Fabienne D Simonis, Marcus J Schultz, Jennifer C Goodson, Troy SB rowne, Leanlove Navarra, Anna Hunt, Robyn A Hutchison, Mathew B Bailey, Lynette Newby, Colin Mcarthur, Michael Kalkoff, Alex Mcleod, Jonathan Casement, Danielle J Hacking, Finn H Andersen, Merete S Dolva, Jon H Laake, Andreas Barratt-Due, Kim Andre L Noremark, Eldar Søreide, Brit Å Sjøbø, Anne B Guttormsen, Hector HLeon Yoshido, Ronald Zumaran Aguilar, Fredy A Montes Oscanoa, Alain U Alisasis, Joanne B Robles, Rossini Abbie B Pasanting-Lim, Beatriz C Tan, Pawel Andruszkiewicz, Karina Jakubowska, Cristina M Cox, António M Alvarez, Bruno S Oliveira, Gustavo M Montanha, Nelson C Barros, Carlos S Pereira, António M Messias, Jorge M Monteiro, Ana M Araujo, Nuno T Catorze, Susan M Marum, Maria J Bouw, Rui M Gomes, Vania A Brito, Silvia Castro, Joana M Estilita, Filipa M Barros, Isabel M Serra, Aurelia M Martinho, Dana R Tomescu, Alexandra Marcu, Ovidiu H Bedreag, Marius Papurica, Dan E Corneci, Silvius Ioan Negoita, Evgeny Grigoriev, Alexey I Gritsan, Andrey A Gazenkampf, Ghaleb Almekhlafi, Mohamad M Albarrak, Ghanem M Mustafa, Khalid A Maghrabi, Nawal Salahuddin, Tharwat M Aisa, Ahmed S Al Jabbary, Edgardo Tabhan, Yaseen M Arabi, Olivia A Trinidad, Hasan M Al Dorzi, Edgardo E Tabhan, Stefan Bolon, Oliver Smith, Jordi Mancebo, Hernan Aguirre-Bermeo, Juan C Lopez-Delgado, Francisco Esteve, Gemma Rialp, Catalina Forteza, Candelaria De Haro, Antonio Artigas, Guillermo M Albaiceta, Sara De Cima-Iglesias, Leticia Seoane-Quiroga, Alexandra Ceniceros-Barros, Antonio L Ruiz-Aguilar, Luis M Claraco-Vega, Juan Alfonso Soler, Maria del Carmen Lorente, Cecilia Hermosa, Federico Gordo, Miryam Prieto-González, Juan B López-Messa, Manuel P Perez, Cesar P Pere, Raquel Montoiro Allue, Ferran Roche-Campo, Marcos Ibañez-Santacruz, Susana Temprano, Maria C Pintado, Raul De Pablo, Pilar Ricart Aroa Gómez, Silvia Rodriguez Ruiz, Silvia Iglesias Moles, Mª Teresa Jurado, Alfons Arizmendi, Enrique A Piacentini, Nieves Franco, Teresa Honrubia, Meisy Perez Cheng, Elena Perez Losada, Javier Blanco, Luis J Yuste, Cecilia Carbayo-Gorriz, Francisca G Cazorla-Barranquero, Javier G Alonso, Rosa S Alda, Ángela Algaba, Gonzalo Navarro, Enrique Cereijo, Esther Diaz-Rodriguez, Diego Pastor Marcos, Laura Alvarez Montero, Luis Herrera Para, Roberto Jimenez Sanchez, Miguel Angel Blasco Navalpotro, Ricardo Diaz Abad, Raquel Montiel González, Dácil Parrilla Toribio, Alejandro G Castro, Maria Jose D Artiga, Oscar Penuelas, Tomas P Roser, Moreno F Olga, Elena Gallego Curto, Rocío Manzano Sánchez, Vallverdu P Imma, Garcia M Elisabet, Laura Claverias, Monica Magret, Ana M Pellicer, Lucia L Rodriguez, Jesús Sánchez-Ballesteros, Ángela González-Salamanca, Antonio G Jimenez, Francisco P Huerta, Juan Carlos J Sotillo Diaz, Esther Bermejo Lopez, David D Llinares Moya, Alec A Tallet Alfonso, Palazon Sanchez Eugenio Luis, Palazon Sanchez Cesar, Sánchez I Rafael, Corcoles G Virgilio, Noelia N Recio, Richard O Adamsson, Christian C Rylander, Bernhard Holzgraefe, Lars M Broman, Joanna Wessbergh, Linnea Persson, Fredrik Schiöler, Hans Kedelv, Anna Oscarsson Tibblin, Henrik Appelberg, Lars Hedlund, Johan Helleberg, Karin E Eriksson, Rita Glietsch, Niklas Larsson, Ingela Nygren, Silvia L Nunes, Anna-Karin Morin, Thomas Kander, Anne Adolfsson, Lise Piquilloud, Hervé O Zender, Corinne Leemann-Refondini, Souheil Elatrous, Slaheddine Bouchoucha, Imed Chouchene, Islem Ouanes, Asma Ben Souissi, Salma Kamoun, Oktay Demirkiran, Mustafa Aker, Emre Erbabacan, Ilkay Ceylan, Nermin Kelebek Girgin, Menekse Ozcelik, Necmettin Ünal, Basak Ceyda Meco, Onat O Akyol, Suleyman S Derman, Barry Kennedy, Ken Parhar, Latha Srinivasa, Lia McNamee, Danny McAuley, Jack Steinberg, Phil Hopkins, Clare Mellis, Frank Stansil, Vivek Kakar, Dan Hadfield, Christine Brown, Andre Vercueil, Kaushik Bhowmick, Sally K Humphreys, Andrew Ferguson, Raymond Mckee, Ashok S Raj, Danielle A Fawkes, Philip Watt, Linda Twohey, Rajeev RJha Matthew Thomas, Alex Morton, Varsha Kadaba, Mark J Smith, Anil P Hormis, Santhana G Kannan, Miriam Namih, Henrik Reschreiter, Julie Camsooksai, Alek Kumar, Szabolcs Rugonfalvi, Christopher Nutt, Orla Oneill, Colette Seasman, Ged Dempsey, Christopher J Scott, Helen E Ellis, Stuart Mckechnie, Paula J Hutton, Nora N Di Tomasso, Michela N Vitale, Ruth O Griffin, Michael N Dean, Julius H Cranshaw, Emma L Willett, Nicholas Ioannou, Sarah Gillis, Peter Csabi, Rosaleen Macfadyen, Heidi Dawson, Pieter D Preez, Alexandra J Williams, Owen Boyd, Laura Ortiz-Ruiz De Gordoa, Jon Bramall, Sophie Symmonds, Simon K Chau, Tim Wenham, Tamas Szakmany, Piroska Toth-Tarsoly, Katie H Mccalman, Peter Alexander, Lorraine Stephenson, Thomas Collyer, Rhiannon Chapman, Raphael Cooper, Russell M Allan, Malcolm Sim, David W Wrathall, Donald A Irvine, Kim S Zantua, John C Adams, Andrew J Burtenshaw, Gareth P Sellors, Ingeborg D Welters, Karen E Williams, Robert J Hessell, Matthew G Oldroyd, Ceri E Battle, Suresh Pillai, Istvan Kajtor, Mageswaran Sivashanmugave, Sinead C Okane, Adrian Donnelly, Aniko D Frigyik, Jon P Careless, Martin M May, Richard Stewart, TJohn Trinder, Samantha J Hagan, Matt P Wise, Jade M Cole, Caroline C MacFie, Anna T Dowling, Javier Hurtado, Nicolás Nin, Javier Hurtado, Edgardo Nuñez, Gustavo Pittini, Ruben Rodriguez, María C Imperio, Cristina Santos, Ana G França, Alejandro Ebeid, Alberto Deicas, Carolina Serra, Aditya Uppalapati, Ghassan Kamel, Valerie M Banner-Goodspeed, Jeremy R Beitler, Satyanarayana Reddy Mukkera, Shreedhar Kulkarni, Jarone Lee, Tomaz Mesar, John O Shinn III, Dina Gomaa, Christopher Tainter, Tomaz Mesar, RAdams Cowley, Dale J Yeatts, Jessica Warren, Michael J Lanspa, Russel R Miller, Colin K Grissom, Samuel M Brown, Philippe R Bauer, Ryan J Gosselin, Barrett T Kitch, Jason E Cohen, Scott H Beegle, Renaud M Gueret, Aiman Tulaimat, Shazia Choudry, William Stigler, Hitesh Batra, Nidhi G Huff, Keith D Lamb, Trevor W Oetting, Nicholas M Mohr, Claine Judy, Shigeki Saito, Fayez M Kheir, Adam B Schlichting, Angela Delsing, Mary Elmasri, Daniel R Crouch, Dina Ismail, Thomas C Blakeman, Kyle R Dreyer, Dina Gomaa, Rebecca M Baron, Carolina Quintana Grijalba, Peter C Hou, Raghu Seethala, Imo Aisiku, Galen Henderson, Gyorgy Frendl, Sen-Kuang Hou, Robert L Owens, Ashley Schomer, Vesna Bumbasirevic, Bojan Jovanovic, Maja Surbatovic, Milic Veljovic, PRoVENT–COVID–, Jesse P. van Akkeren, Anna Geke Algera, Cheetel K. Algoe, Rombout B. van Amstel, Onno L. Baur, Pablo van de Berg, Alida E. van den Berg, Dennis C. J. J. Bergmans, Dido I. van den Bersselaar, Freke A. Bertens, Alexander J. G. H. Bindels, Milou M. de Boer, Sylvia den Boer, Leonoor S. Boers, Margriet Bogerd, Lieuwe D. J. Bos, Michela Botta, Jennifer S. Breel, Hendrik de Bruin, Sanne de Bruin, Caro L. Bruna, Laura A. Buiteman-Kruizinga, Olaf L. Cremer, Rogier M. Determann, Willem Dieperink, Dave A. Dongelmans, Hildegard S. Franke, Michal S. Galek-Aldridge, Mart J. de Graaff, Laura A. Hagens, Jasper J. Haringman, Sebastiaan T. van der Heide, Pim L. J. van der Heiden, Nanon F. L. Heijnen, Stephan J. P. Hiel, Lotte L. Hoeijmakers, Liselotte Hol, Markus W. Hollmann, Marga E. Hoogendoorn, Janneke Horn, Robrecht van der Horst, Evy L. K. Ie, Dimitri P. Ivanov, Nicole Juffermans, Eline Kho, Eline S. de Klerk, Ankie W. M. M. Koopman-van Gemert, Matty Koopmans, Songul Kucukcelebi, Michael A. Kuiper, Dylan W. de Lange, Niels van Mourik, Sunny G. L. H. Nijbroek, Marisa Onrust, Evelien A. N. Oostdijk, Frederique Paulus, Charlotte J. Pennartz, Janesh Pillay, Luigi Pisani, Ilse M. Purmer, Thijs C. D. Rettig, Jan-Paul Roozeman, Michiel T. U. Schuijt, Marcus J. Schultz, Ary Serpa Neto, Mengalvio E. Sleeswijk, Marry R. Smit, Peter E. Spronk, Willemke Stilma, Aart C. Strang, Anissa M. Tsonas, Pieter R. Tuinman, Christel M. A. Valk, Felicia L. Veen-Schra, Lars I. Veldhuis, Patricia van Velzen, Ward H. van der Ven, Alexander P. J. Vlaar, Peter van Vliet, Peter H. J. van der Voort, Louis van Welie, Henrico J. F. T. Wesselink, Hermien H. van der Wier-Lubbers, Bas van Wijk, Tineke Winters, Wing Yi Wong, Arthur R. H. van Zanten, EPICCoV–, Juliana C. Ferreira, Yeh-Li Ho, Bruno A. M. P. Besen, Luiz M. S. Malbuisson, Leandro U. Taniguchi, Pedro Mendes IV, Eduardo L. V. Costa, Marcelo Park, Renato Daltro-Oliveira, Roberta M. L. Roepke, João M. Silva, Maria José C. Carmona, Carlos Roberto Ribeiro Carvalho, Adriana Hirota, Alberto Kendy Kanasiro, Alessandra Crescenzi, Amanda Coelho Fernandes, Anna Miethke-Morais, Arthur Petrillo Bellintani, Artur Ribeiro Canasiro, Bárbara Vieira Carneiro, Beatriz Keiko Zanbon, Bernardo Pinheiro De Senna Nogueira Batista, Bianca Ruiz Nicolao, Bruno Adler Maccagnan Pinheiro Besen, Bruno Biselli, Bruno Rocha De Macedo, Caio Machado Gomes De Toledo, Carlos Eduardo Pompilio, Carlos Roberto Ribeiro De Carvalho, Caroline Gomes Mol, Cassio Stipanich, Caue Gasparotto Bueno, Cibele Garzillo, Clarice Tanaka, Daniel Neves Forte, Daniel Joelsons, Daniele Robira, Eduardo Leite Vieira Costa, Elson Mendes Da Silva Júnior, Fabiane Aliotti Regalio, Gabriela Cardoso Segura, Gustavo Brasil Marcelino, Giulia Sefrin Louro, Yeh-Li Ho, Isabela Argollo Ferreira, Jeison de Oliveira Gois, Joao Manoel Da Silva Junior, Jose Otto Reusing Junior, Julia Fray Ribeiro, Juliana Carvalho Ferreira, Karine Vusberg Galleti, Katia Regina Silva, Larissa Padrao Isensee, Larissa dos Santos Oliveira, Leandro Utino Taniguchi, Leila Suemi Letaif, Lígia Trombetta Lima, Lucas Yongsoo Park, Lucas Chaves Netto, Luciana Cassimiro Nobrega, Luciana Haddad, Ludhmila Hajjar, Luiz Marcelo Malbouisson, Manuela Cristina Adsuara Pandolfi, Marcelo Park, Maria José Carvalho Carmona, Maria Castilho Prandini H De Andrade, Mariana Moreira Santos, Matheus Pereira Bateloche, Mayra Akimi Suiama, Mayron Faria de Oliveira, Mayson Laercio Sousa, Michelle Louvaes, Natassja Huemer, Pedro Mendes, Paulo Ricardo Gessolo Lins, Pedro Gaspar Dos Santos, Pedro Ferreira Paiva Moreira, Renata Mello Guazzelli, Renato Batista Dos Reis, Renato Daltro De Oliveira, Roberta Muriel Longo Roepke, Rodolpho Augusto De Moura Pedro, Rodrigo Kondo, Samia Zahi Rached, Sergio Roberto Silveira Da Fonseca, Thais Sousa Borges, Thalissa Ferreira, Vilson Cobello Junior, Vivian Vieira Tenório Sales, Willaby Serafim Cassa Ferreira, CIBERESUCICOVID–, Rafael Mañez, Felipe Rodríguez de Castro, María Mora Aznar, Mateu Torres, María Martinez, Cynthia Alegre, Sofía Contreras, Javier Trujillano, Montse Vallverdú, Miguel León, Mariona Badía, Begoña Balsera, Lluís Servià, Judit Vilanova, Silvia Rodríguez, Neus Montserrat, Silvia Iglesias, Javier Prados, Sula Carvalho, Mar Miralbés, Josman Monclou, Gabriel Jiménez, Jordi Codina, Estela Val, Pablo Pagliarani, Jorge Rubio, Dulce Morales, Andrés Pujol, Àngels Furro, Beatriz García, Gerard Torres, Javier Vengoechea, David de Gozalo Calvo, Jessica González, Silvia Gomez, Lorena Forcelledo Espina, Emilio García Prieto, Paula Martín Vicente, Cecilia del Busto Martínez, María Aguilar Cabello, Carmen Eulalia Martínez Fernández, María Luisa Blasco Cortés, Ainhoa Serrano Lázaro, Mar Juan Díaz, María Teresa Bouza Vieiro, Inés Esmorís Arijón, David Campi Hermoso, Rafaela Nogueras Salinas, Teresa Farre Monjo, Ramon Nogue Bou, Gregorio Marco Naya, Núria Ramon Coll, Juan Carlos Montejo-González, Gloria Renedo Sanchez-Giron, Juan Bustamante-Munguira, Ramon Cicuendez Avila, Nuria Mamolar Herrera, Alexander Agrifoglio, Lucia Cachafeiro, Emilio Maseda, Albert Figueras, Maria Teresa Janer, Laura Soliva, Marta Ocón, Luisa Clar, JIgnacio Ayestarán, Sandra Campos Fernández, Eva Forcadell-Ferreres, Immaculada Salvador-Adell, Neus Bofill, Berta Adell-Serrano, Josep Pedregosa Díaz, Núria Casacuberta-Barberà, Luis Urrelo-Cerrón, Àngels Piñol-Tena, Ferran Roche-Campo, Pablo Ryan Murúa, Covadonga Rodríguez Ruíz, Laura Carrión García, Juan ILazo Álvarez, Desire Macias Guerrero, Daniel Tognetti, Carlos García Redruello, David Mosquera Rodríguez, Eva María Menor Fernández, Sabela Vara Adrio, Vanesa Gómez Casal, Marta Segura Pensado, María Digna Rivas Vilas, Amaia García Sagastume, Raul de Pablo Sánchez, David Pestaña Laguna, Tommaso Bardi, Carmen Gómez Gonzalez, Maria Luisa Gascón Castillo, José Garnacho-Montero, Joan Ramon Masclans, Ana Salazar Degracia, Judit Bigas, Rosana Muñoz-Bermúdez, Clara Vilà-Vilardel, Francisco Parrilla, Irene Dot, Ana Zapatero, Yolanda Díaz, María Pilar Gracia, Purificación Pérez, Andrea Castellví, Cristina Climent, Lidia Serra, Laura Barbena, Iosune Cano, Alba Herraiz, Pilar Marcos, Laura Rodríguez, Maria Teresa Sariñena, Ana Sánchez, Juan Fernando Masa Jimenez, Gemma Gomà, Mercedes Ibarz, Diego De Mendoza, Enric Barbeta, Victoria Alcaraz-Serrano, Joan Ramon Badia, Manuel Castella, Leticia Bueno, Laia Fernandez Barat, Catia Cillóniz, Pamela Conde, Javier Fernández, Albert Gabarrus, Karsa Kiarostami, Alexandre López-Gavín, Cecilia L Mantellini, Carla Speziale, Nil Vázquez, Hua Yang, Minlan Yang, Carlos Ferrando, Pedro Castro, Marta Arrieta, Jose Maria Nicolas, Rut Andrea, Marta Barroso, Sergio Álvarez, Dario Garcia-Gasulla, Adrián Tormos, Cesar Aldecoa, Rubén Herrán-Monge, José Ángel Berezo García, Pedro Enríquez Giraudo, Pablo Cardinal Fernández, Alberto Rubio López, Orville Báez Pravia, Leire Pérez Bastida, Antonjo Alvarez Ruiz, Anna Parera Pous, Ana López Lago, Eva Saborido Paz, Patricia Barral Segade, Manuel Valledor Mendez, Luciano Aguilera, Esther López-Ramos, Ángela Leonor Ruiz-García, Belén Beteré, Rafael Blancas, Cristina Dólera, Gloria Perez Planelles, Enrique Marmol Peis, Maria Dolores Martinez Juan, Miriam Ruiz Miralles, Eva Perez Rubio, Maria Van der Hofstadt Martin-Montalvo, Tatiana Villada Warrington, Sara Guadalupe Moreno Cano, Federico Gordo, Basilisa Martinez Palacios, Maria Teresa Nieto, Sergio Ossa, Ana Ortega, Miguel Sanchez, Bitor Santacoloma, SATI–COVID–19–investigators, Elisa Estenssoro, Arnaldo Dubin, Cecilia Inés Loudet, Fernando Ríos, Vanina Siham Kanoore Edul, Gustavo Plotnikow, Rosa Reina Macarena Andrian, Julián Ivacachi, Ignacio Romero, Carla Garay, Damián Piezny, Judith Sagardía, Marco Bezzi, Silvia Borello, Verónica Mandich, Daniel Chiacchiara, Carla Groer, Constanza García Almirón, Ana Kovac, Sebastián Torres, Cristian Cesio, Cristina Orlandi, Rosana Hernández, Paolo Nahuel Rubatto Birri, Matías Mugno, Florencia Valenti, Raúl Alejandro Gómez, Eleonora Cunto, Viviana Chediack, María Gabriela Sáenz, Cecilia Marchena, Norberto Tiribelli, María Guayma, Vanina Aphalo, Daniela Vazquez, Yasmin Saad, Diego Sanchez, Federico Iglesias, Pablo Casteluccio, Bernardo Lattanzio, Sebastián Eiguren, Diego Noval, Sebastián Fredes, Gabriela Izzo, Horacio Cabrera, Mario Pozo, Santiago Sac, Nicolás Tornatore, Julia Sakugawa, Celeste Villafañe, Antonio Di Sibio, Patricio Maskin, Pablo Rodríguez, Nicolás Nihany, Mariela Mogadouro, Fernando Pálizas, Emiliano Cornú, Mariano Esperatti, Juan Manuel Pintos, Gustavo Badariotti, Gonzalo Echevarría, Ana María Mazzola, Cecilia Giuggia, Nahuel Dargains, Alejandra Turano, Florencia Pugliese, Marcos Zec Baskarad, Mariana Chamadoira, Juan Carlos Medina, Marina Búsico, Fernando Villarejo, Hugo Collazos, Tania Huanca, Juan Carlos Pendino, Lionel Talamonti, Fernando Skrzypiec, Claudia Tascón, Gabriela Genovese, Hugo Alul, Agustina Zavattieri, Ana Julieta Herrera, Norma Rosales, María Gabriela Quintana, Alejandro Risso Vazquez, Martín Lugaro, Eduardo Díaz Rousseaux, Marcelo Falcone, Fernando Kurban, Matías Cini, Graciela Zakalik, Carlos Pellegrini, Gabriela Fernández, Juan Pablo Sottile, Sol Barrios, Orlando Hamada, Verónica Mendiluce, Darío Villalba, Florencia Sacco, Vito Mezzina, Carlos Servin, Mónica Quinteros, Hernán Nuñez, María Luz Campassi, David Banegas, Carina Balasini, Victoria Leiva, Franco Maicol, Gustavo Domeniconi, Verónica Vilaseca, Alejandra Barrientos, Florencia Larocca, Liliana Kumar, Rosa Luna, Martín Deheza Lonardi, Agustina Oholeguy, Joaquín Carnero Echegaray, Carla Marazzi, Plácido Helca Regis, Federico Rópolo, Adrián Bobadilla, Vivian Thomas, Nydia Funes Nelson, Cintia Villavicencio, Pedro Machare, Norma Aramayo, Cecilia González, Mariano Ferriccioni, Judith Bergesio

**Affiliations:** 1 Department of Intensive Care, Amsterdam University Medical Centers, Location ‘AMC’, Meibergdreef 9, 1105 AZ Amsterdam, The Netherlands; 2 Department of Intensive Care, Rode Kruis Ziekenhuis, Beverwijk, The Netherlands; 3 Department of Emergency Medicine, Hospital das Clinicas HCFMUSP, Faculdade de Medicina, Universidade de São Paulo, São Paulo, Brazil; 4 Department of Intensive Care, Hospital Israelita Albert Einstein, São Paulo, Brazil; 5 Centre for Medical Sciences (CISMed), University of Trento, Trento, Italy; 6 Department of Anesthesia and Intensive Care, Santa Chiara Hospital, APSS Trento, Trento, Italy; 7 Department of Intensive Care, Hospital Interzonal de Agudos General San Martin La Plata, Buenos Aires, Argentina; 8 Interdepartmental Division of Critical Care Medicine, University of Toronto, Toronto, ON Canada; 9 Department of Pulmonology, Instituto Do Coracao (InCor), Hospital das Clinicas HCFMUSP, Faculdade de Medicina, Universidade de Sao Paulo, São Paulo, Brazil; 10 Department of Intensive Care, AC Camargo Cancer Center, São Paulo, Brazil; 11 Brazilian Research in Intensive Care Network (BRICNet), São Paulo, Brazil; 12 Department of Anaesthesiology and Intensive Care, Galway University Hospital, Saolta Hospital Group, Galway, Ireland; 13 School of Medicine, University of Galway, Galway, Ireland; 14 Department of Intensive Care, Multidisciplinary Intensive Care Research Organization (MICRO), St James’ Hospital, Dublin, Ireland; 15 Department of Intensive Care, Hospital Clínic de Barcelona, Barcelona, Spain; 16 Departement of Pulmonology, Institut d’Investigacions Biomèdiques August Pi I Sunyer (IDIBAPS), Hospital Clínic de Barcelona, Barcelona, Spain; 17 Centro de Investigación Biomédica en Red en Enfermedades Respiratorias (CIBERES), Institute of Health Carlos III, Madrid, Spain; 18 Equipe d’Epidémiologie Respiratoire Integrative, Université Paris–Saclay, Paris, France; 19 Service de Médecine Intensive-Réanimation, DMU CORREVE, FHU SEPSIS, Groupe de Recherche Clinique CARMAS, Hôpital de Bicêtre, Paris, France; 20 Department of Intensive Care, Hospital Universitario de Getafe, Getafe, Spain; 21 Fondazione IRCCS Ca’ Granda Ospedale Maggiore Policlinico, Milan, Italy; 22 Department of Anesthesia and Intensive Care, Miulli Regional Hospital, Acquaviva Delle Fonti, Italy; 23 Australian and New Zealand Intensive Care Research Centre (ANZIC–RC), Monash University, Melbourne, Australia; 24 Mahidol–Oxford Tropical Medicine Research Unit (MORU), Mahidol University, Bangkok, Thailand; 25 Nuffield Department of Medicine, University of Oxford, Oxford, UK; 26 Department of Anesthesia, General Intensive Care and Pain Management, Division of Cardiothoracic and Vascular Anesthesia & Critical Care Medicine, Medical University of Vienna, Vienna, Austria; 27 Laboratory of Experimental Intensive Care & Anaesthesiology (L·E·I·C·A), Amsterdam UMC, Location AMC, Amsterdam, The Netherlands; 28 University of Barcelona, Barcelona, Spain; 29 Catalan Institution for Research and Advanced Studies (ICREA), Barcelona, Spain; 30 Center of Expertise Urban Vitality, Faculty of Health, Amsterdam University of Applied Sciences, Amsterdam, The Netherlands; 31 Department of Anaesthesiology, Amsterdam UMC, Location AMC, Amsterdam, The Netherlands

**Keywords:** Acute respiratory distress syndrome, ARDS, COVID–19, Critical care, Mechanical ventilation, Ventilation management

## Abstract

**Background:**

Ventilation management may differ between COVID–19 ARDS (COVID–ARDS) patients and patients with pre–COVID ARDS (CLASSIC–ARDS); it is uncertain whether associations of ventilation management with outcomes for CLASSIC–ARDS also exist in COVID–ARDS.

**Methods:**

Individual patient data analysis of COVID–ARDS and CLASSIC–ARDS patients in six observational studies of ventilation, four in the COVID–19 pandemic and two pre–pandemic. Descriptive statistics were used to compare epidemiology and ventilation characteristics. The primary endpoint were key ventilation parameters; other outcomes included mortality and ventilator–free days and alive (VFD–60) at day 60.

**Results:**

This analysis included 6702 COVID–ARDS patients and 1415 CLASSIC–ARDS patients. COVID–ARDS patients received lower median V_T_ (6.6 [6.0 to 7.4] vs 7.3 [6.4 to 8.5] ml/kg PBW; *p* < 0.001) and higher median PEEP (12.0 [10.0 to 14.0] vs 8.0 [6.0 to 10.0] cm H_2_O; *p* < 0.001), at lower median ΔP (13.0 [10.0 to 15.0] vs 16.0 [IQR 12.0 to 20.0] cm H_2_O; *p* < 0.001) and higher median Crs (33.5 [26.6 to 42.1] vs 28.1 [21.6 to 38.4] mL/cm H_2_O; *p* < 0.001). Following multivariable adjustment, higher ΔP had an independent association with higher 60–day mortality and less VFD–60 in both groups. Higher PEEP had an association with less VFD–60, but only in COVID–ARDS patients.

**Conclusions:**

Our findings show important differences in key ventilation parameters and associations thereof with outcomes between COVID–ARDS and CLASSIC–ARDS.

**Trial registration:**

Clinicaltrials.gov (identifier NCT05650957), December 14, 2022.

**Supplementary Information:**

The online version contains supplementary material available at 10.1186/s12931-024-02910-2.

## Background

The high numbers of patients who needed invasive ventilation early in the unprecedented pandemic of coronavirus disease 2019 (COVID–19) has led to numerous studies of epidemiology, ventilation management and outcomes in patients with acute respiratory distress syndrome (ARDS) related to an infection with SARS–CoV–2. COVID–19 ARDS would differ from ARDS before the pandemic (CLASSIC–ARDS) in several aspects [1, 2], and different phenotypes have even been suggested [3, 4].

The number of studies that directly compared ventilation management of COVID–ARDS with CLASSIC–ARDS is limited [5, 6]. It remains uncertain whether practice of invasive ventilation in COVID–ARDS patients really differed from that in CLASSIC–ARDS patients. It is also unknown whether associations of certain aspects of ventilation with outcomes found in CLASSIC–ARDS also exist in COVID–ARDS. This would have serious implications on how to set the ventilator in the two patient groups, as then certain recommendations in guidelines for ventilation in CLASSIC–ARDS may not apply in COVID–ARDS [7].

We performed an analysis of a conveniently–sized database that pooled the data of individual patients of six observational ventilation studies, four of which were conducted in the COVID–19 pandemic and two pre–pandemic, to compare epidemiology, ventilator management and associations of ventilation characteristics and outcome between COVID–ARDS and CLASSIC–ARDS patients. To have comparable patient groups, we only selected patients with ARDS from a respiratory infection from the two pre–pandemic studies. We hypothesized that key ventilator parameters would be different between the two groups, and used multivariable analyses to determine associations with outcomes.

## Methods

### Study design and participants

This is a meta–analysis using the individual patient data of patients in six preselected large observational studies focusing on a diverse representation of epidemiological features and ventilation management in both COVID–19 and pre–pandemic ARDS. The six studies were selected because they all contained detailed data on epidemiological features, ventilation data, and outcomes, originating from various regions worldwide, both in resource–limited and resource–rich settings.

The corresponding authors of the original studies accepted the invitation, after which the data dictionaries of the studies were compared to check whether the data could be harmonized. Then, the databases were transferred after local approval and agreement on the analysis plan of the current investigation.

The two pre–pandemic studies were the national ‘Epidemiology of Respiratory Insufficiency in Critical Care’ study (ERICC) conducted in 2011 in Brazil [8], and the international ‘Large Observational Study to UNderstand the Global Impact of Severe Acute Respiratory FailurE’ study (LUNG SAFE) conducted in 2014 in 50 countries worldwide [9]. All four studies were conducted during the COVID–19 pandemic, ranging from March 2020 to 2021 and included: the national ‘Practice of Ventilation in COVID–19 patients’ study (PRoVENT–COVID) from The Netherlands [10], the national ‘EPIdemiology of Critical COVID–19’ study (EPICCoV) from Brazil [11, 12], the national ‘Centro de Investigación Biomédica en Red Enfermedades Respiratorias COVID–19 study’ (CIBERESUCICOVID) from Spain [13], and the national ‘Sociedad Argentina de Terapia Intensiva–COVID–19 study’ (SATI–COVID–19) from Argentina [14].

The study protocols of the original studies were approved by Institutional Review Boards if applicable, and need for individual patient informed consent was waived for all studies due to their observational designs. Details of all studies can be found in the original publications [8–10, 12–14]. We invited the corresponding investigators of the original studies to provide us the case report forms and data dictionaries, and the data of all patients. The creation of the pooled database did not require additional ethical approval. The databases of the original studies were harmonised using the case report forms and data dictionaries, and finally merged. This current analysis is registered at clinicaltrials.gov (study identifier NCT05650957), and its statistical analysis plan was finalized before cleaning and closing of the database.

Patients in the merged database were eligible for participation in this current analysis if: (1) aged 18 years or higher; (2) having received invasive ventilation within the first 48 h of ICU admission, regardless of its duration; and (3) fulfilling the Berlin definition of ARDS. We excluded CLASSIC–ARDS patients when ARDS was reported not to be caused by a respiratory infection.

### Data available for merging

The following baseline and demographic variables were available for merging into the new database—sex, age, body weight and height, comorbidities including hypertension and cardiac failure, chronic obstructive pulmonary disease (COPD), diabetes mellitus, kidney failure, liver failure, and cancer, date of hospital and intensive care unit (ICU) admission, and disease severity scores, including the Simplified Acute Physiology Score (SAPS) II at ICU admission and a daily Sequential Organ Failure Assessment (SOFA) scores.

Collected ventilation variables were––mode of ventilation, tidal volume (V_T_), positive end–expiratory pressure (PEEP), fraction of inspired oxygen (FiO_2_), respiratory rate (RR), peak pressure (Ppeak) in volume–controlled ventilation and plateau pressure (Pplat) in pressure–controlled ventilation, blood gas analyses results, and adjunctive therapies to improve oxygenation in case of refractory hypoxaemia. The first available measurement of the day was used. If multiple measurements were taken on the same day, we selected earliest one.

The dynamic driving pressure (ΔP) was calculated by subtracting PEEP from the maximum airway pressure [15, 16]. Respiratory system compliance (Crs) was calculated by dividing V_T_ by ΔP. MP was calculated using the power Eq. (17), wherein MP (J/min) = 0.098 * V_T_ * RR * (Ppeak − 0.5 * ΔP) [17]; a modified power equation was used if no Ppeak was available 0.098 * V_T_ * RR * (Pplat − 0.5 * ΔP) [16]. The ventilatory ratio was calculated as (minute ventilation * PaCO_2_)/(predicted bodyweight * 100 * 37.5) [18]. The number of ventilator–free days at day 60 (VFD–60) was calculated by subtracting the number of calendar days a patient received invasive ventilation up to the day of successful extubation from 60, similar to the method used for calculating VFD–28. Patients that died before or at day 60 received zero VFD–60 [19, 20].

The following follow–up data were available for merging—last day of ventilation, tracheostomy use, last day in ICU and hospital, and life status at day 60.

### Endpoints

The primary endpoint of this analysis was a combination of the following key ventilation characteristics as done before [10]—V_T_, PEEP, ΔP, and Crs. Secondary outcomes were other ventilator parameters, the use of prone positioning, muscle paralysis or extracorporeal membrane oxygenation, and 60–day mortality and the number of VFD–60.

### Power analysis

We did not perform a formal power analysis; instead, the number of available patients served as the sample size.

### Statistical analysis

Baseline demographics were compared using Fisher’s exact tests for categorical variables and Wilcoxon rank–sum tests for continuous variables. Continuous distributed variables are presented as medians and interquartile ranges, categorical variables are presented as frequencies and proportions.

The first day a patient received invasive ventilation and the first full calendar day were combined into ‘day 1’, the next day was designated as ‘day 2’. Information on missing values for each ventilation parameters and other variable can be found in the Supplementary Material (eTable 1). Only SOFA scores were available for all patients, therefore, we chose to only report these instead of other severity scores.

To compare ventilation characteristics between COVID–ARDS and CLASSIC–ARDS patients, a Wilcoxon rank–sum test was used. Cumulative distribution plots were constructed to visualize cumulative distribution frequencies of each ventilation variable or parameter, wherein vertical dotted lines represent broadly accepted safety cutoffs for each variable, and horizontal dotted lines show the respective proportion of patients reaching that cutoff.

As a post–hoc analysis to identify whether V_T_, PEEP and ΔP have independent associations with 60–day mortality and the number of VFD–60, a multivariable mixed–effects model with centre as random effect was performed. A linear mixed–effects model was used for the number of VFD–60 and a logistic mixed–effects model for 60–day mortality.

The following covariates, with a known or suspected association with these two outcomes were included in the model, based on clinical relevance: (1) PaO_2_/FiO_2_; and (2) demographic variables, including sex, age, BMI, history of heart failure, COPD, diabetes mellitus, kidney failure, liver failure and cancer.

In this mixed model analysis, when a covariate exhibited more than 10% missing data, we utilized multiple imputation techniques implemented through the MICE package in R. The model was checked for collinearity using variance–inflation factors, wherein a variance–inflation factor < 5 was deemed acceptable. The variance–inflation factor was < 2 for all included variables in our model.

The estimate refers to the average effect of the ventilation parameter, i.e., V_T_, PEEP or ΔP on the outcome of interest, i.e., 60–day mortality and VFD–60 while controlling for the other variables in the model. A positive estimate indicates that an increase in the predictor variable tends to lead to a corresponding increase in the response variable, indicating a proportional relationship between them. Conversely, a negative estimate suggests that an increase in the predictor variable tends to result in a decrease in the response variable, indicating an inverse proportional relationship between them.

All analyses were conducted in R v.4.0.3 (R Foundation for Statistical Computing, Vienna, Austria). A *p* value < 0.05 was considered statistically significant.

## Results

**Fig. 1:**
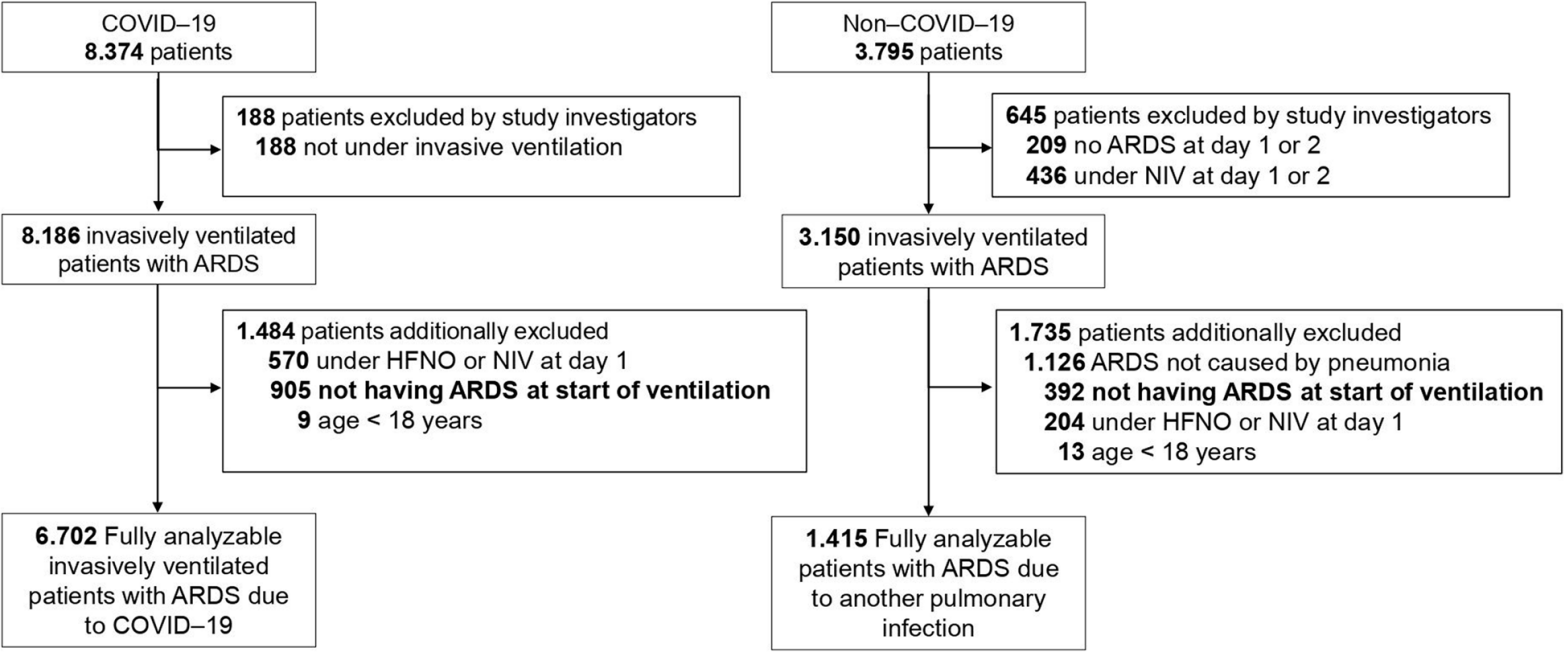
Flowchart of included studies. Abbreviations: ARDS = acute respiratory distress syndrome; COVID–19 = coronavirus disease 2019

We received the individual data of a total of 8374 COVID–ARDS patients and 3795 CLASSIC–ARDS patients (Fig. 1). After exclusion of patients that did not fulfil the Berlin definition of ARDS, patients that did not receive invasive ventilation on the first and second day in the study, and patients included in the two pre–pandemic studies who did not have a respiratory infection as the cause for ARDS, we had 6702 fully–analysable COVID–ARDS patients and 1415 fully–analysable CLASSIC–ARDS. COVID–ARDS patients were more often male, had higher median BMI, a history of diabetes more often, and a history of COPD or chronic kidney disease less often (Table 1). COVID–ARDS patients had lower median SOFA scores, and ARDS severity was more often classified as moderate or severe.

**Table 1:** Patient demographics, Baseline Characteristics and ARDS Severity

	**COVID–ARDS** ***N* = 6.702**	**CLASSIC–ARDS** ***N* = 1.415**	***p***
Demographics			
age, years, median [IQR]	64.0 [55.0 to 71.0]	63.0 [50.0 to 74.0]	0.361
height, cm, median [IQR]	170.0 [163.0 to 175.0]	168.0 [160.0 to 175.0]	< 0.001
weight, kg, median [IQR]	82.0 [74.0 to 95.0]	74.0 [62.0 to 86.0]	< 0.001
male gender, n/N (%)	4.655 (69.5)	859 (60.7)	< 0.001
BMI, kg/m^2^, median [IQR]	28.5 [25.6 to 32.4]	25.8 [22.5 to 30.1]	< 0.001
SOFA score, median [IQR]	6.0 [4.0 to 8.0]	10.0 [7.0 to 12.0]	< 0.001
Comorbidities			
heart failure, N (%)	633 (9.4)	143 (10.1)	0.472
COPD, N (%)	688 (10.3)	323 (22.8)	< 0.001
diabetes, N (%)	1.906 (28.4)	322 (22.8)	< 0.001
chronic kidney disease, N (%)	402 (6.0)	140 (9.9)	< 0.001
liver failure, N (%)	122 (1.8)	53 (3.7)	< 0.001
active neoplasm, N (%)	253 (3.8)	119 (8.4)	< 0.001
ARDS severity categories			< 0.001
mild, N (%)	1.937 (28.9)	362 (25.6)	
moderate, N (%)	3.477 (51.8)	673 (47.6)	
severe, N (%)	1.288 (19.2)	380 (26.9)	

*Abbreviations*: *ARDS* Acute respiratory distress syndrome, *IQR* Interquartile range, *N* Number, *BMI* Body mass Index, *SOFA* Sequential organ failure assessment, *COPD* Chronic obstructive pulmonary disease

**Fig. 2:**
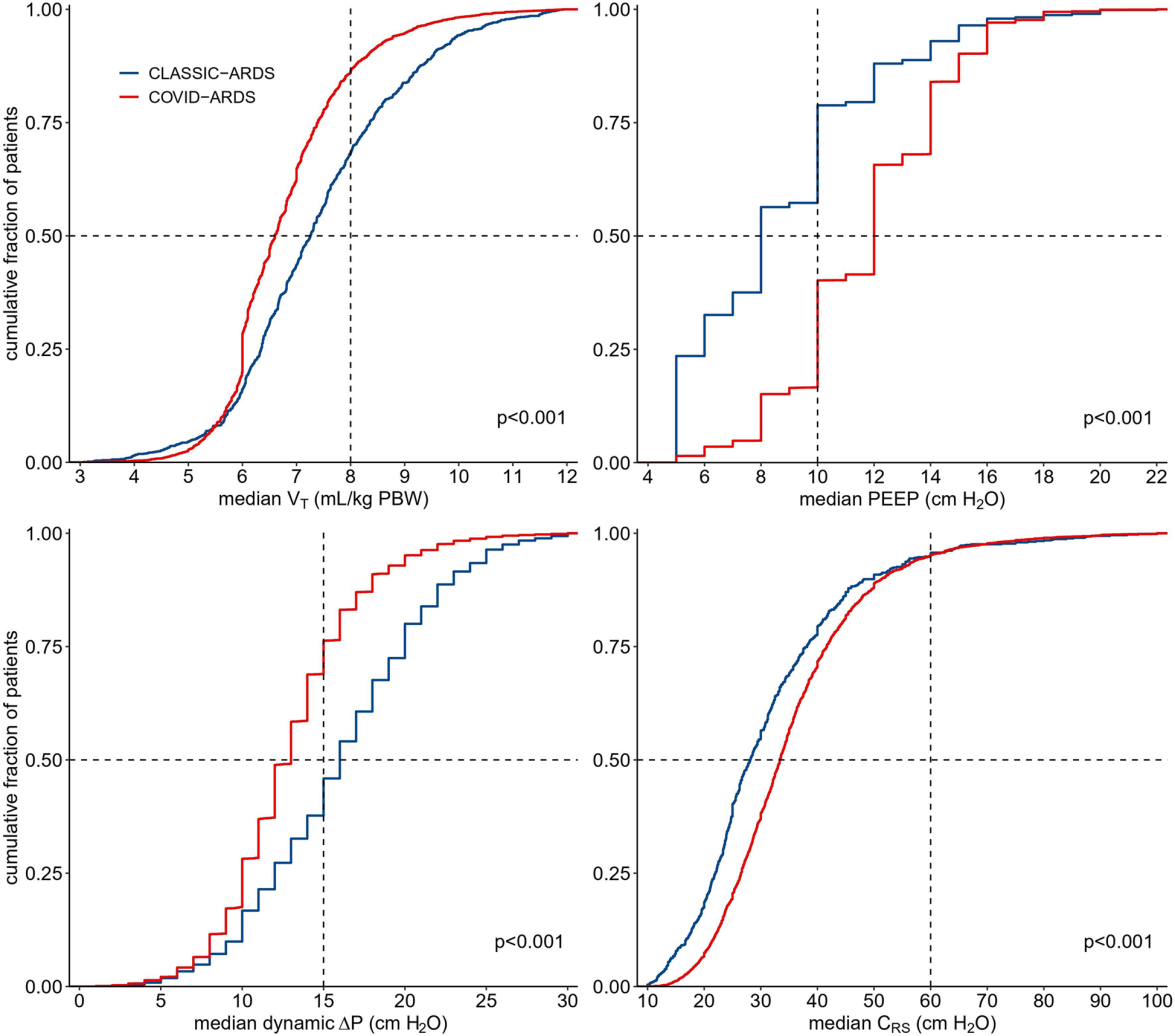
Key ventilation parameters. Cumulative frequency distribution of V_T_, PEEP, ΔP, and respiratory system compliance on the first calendar day for each variable. Vertical dotted lines represent broadly accepted safety cutoffs for each variable, and horizontal dotted lines show the respective proportion of patients reaching that cutoff. Abbreviations: V_T_ = tidal volume; PBW = predicted bodyweight; PEEP = positive end–expiratory pressure; ΔP = driving pressure; C_RS_ = respiratory system compliance

COVID–ARDS patients were ventilated with volume–controlled ventilation more often than CLASSIC–ARDS patients (Table 2) and received ventilation with lower V_T_ (6.6 [6.0 to 7.4] vs 7.3 [6.4 to 8.5] ml/kg PBW; *p* < 0.001), higher PEEP (12.0 [10.0 to 14.0] vs 8.0 [6.0 to 10.0] cm H_2_O; *p* < 0.001), at lower ΔP (13.0 [10.0 to 15.0] vs 16.0 [IQR 12.0 to 20.0] cm H_2_O; *p* < 0.001) and higher Crs (33.5 [26.6 to 42.1] vs 28.1 [21.6 to 38.4] mL/cm H_2_O; *p* < 0.001) (Fig. 2) COVID–ARDS patients received higher PEEP than CLASSIC–ARDS patients at any FiO_2_ level (eFigure 2). Within each group, the ventilation characteristics were not different between day 1 and 2 (eTable 2 and eFigure 1 and 2).

**Table 2:** Ventilation Characteristics, Adjunctive Therapies, Arterial Blood Gas Analysis and Outcomes

	**COVID–ARDS** ***N* = 6.702**	**CLASSIC–ARDS** ***N* = 1.415**	***p***
Ventilation characteristics			
mode of ventilation, N (%)			< 0.001
volume–controlled ventilation	4.689 (70.3)	521 (36.8)	
pressure–controlled ventilation	1.403 (21.0)	493 (34.8)	
pressure–support ventilation	336 (5.0)	143 (10.1)	
other	242 (3.6)	258 (18.2)	
V_T_, mL/kg PBW, median [IQR]	6.6 [6.0 to 7.4]	7.3 [6.4 to 8.5]	< 0.001
< 6 ml/kg PBW	1.464 (24.8)	211 (16.3)	< 0.001
6–8 ml/kg PBW	3.601 (61.2)	645 (50.0)	< 0.001
8–10 ml/kg PBW	698(11.9)	324 (25.1)	< 0.001
> 10 ml/kg PBW	125 (2.1)	102 (7.9)	< 0.001
PEEP, cmH_2_O, median [IQR]	12.0 [10.0 to 14.0]	8.0 [6.0 to 10.0]	< 0.001
< 8 cmH_2_O	325 (4.8)	530 (37.5)	< 0.001
8–12 cmH_2_O	4.075 (60.8)	713 (50.4)	< 0.001
12–16 cmH_2_O	2.101 (31.3)	140 (9.9)	< 0.001
> 16 cmH_2_O	201 (2.9)	32 (2.3)	0.37
P_MAX_, cmH_2_O, median [IQR]	25.5 [21.0 to 30.0]	25.0 [22.0 to 28.0]	0.001
**dynamic** ΔP, cmH_2_O, median [IQR]	13.0 [10.0 to 15.0]	16.0 [12.0 to 20.0]	< 0.001
C_RS_, mL/cmH_2_O, median [IQR]	33.5 [26.6 to 42.1]	28.1 [21.6 to 38.4]	< 0.001
MP, J/min, median [IQR]	16.6 [13.4 to 20.6]	15.2 [11.3 to 19.2]	< 0.001
FiO_2,_ median [IQR]	0.6 [0.5 to 0.9]	0.6 [0.5 to 0.9]	0.017
total RR, breaths per min, median [IQR]	22.0 [20.0 to 25.0]	20.0 [16.0 to 25.0]	< 0.001
ventilatory ratio, median [IQR]	1.75 [1.43 to 2.20]	1.76 [1.35 to 2.27]	0.835
Adjunctive therapies			
prone positioning, N (%)	4.615 (69.2)	144 (10.2)	< 0.001
recruitment manoeuvres, N (%)	1.924 (39.4)	313 (22.1)	< 0.001
ECMO, N (%)	142 (2.5)	56 (4.0)	0.005
tracheostomy, N (%)	2.033 (30.5)	214 (15.1)	< 0.001
neuromuscular blocking agents, N (%)	3.838 (73.9)	350 (24.7)	< 0.001
continuous sedation, N (%)	1.188 (84.0)	1.785 (98.1)	< 0.001
vasopressor use, N (%)	4440 (85.5)	1025 (72.5)	< 0.001
Arterial blood gas analysis			
pH, median [IQR]	7.35 [7.28 to 7.41]	7.34 [7.26 to 7.41]	< 0.001
PaO_2_, mmHg, median [IQR]	80.1 [67.5 to 98.5]	83.6 [68.0 to 105.8]	< 0.001
PaCO_2_, mmHg, median [IQR]	44.25 [38.0 to 52.0]	44.0 [37.0 to 54.0]	0.320
PaO_2_/FiO_2_, median [IQR]	135.0 [94.0 to 184.9]	146.0 [99.4 to 207.3]	< 0.001
Outcomes			
60–day mortality, N (%)	2963 (44.2)	515 (36.4)	< 0.001
VFD–60, median [IQR]	**11.0 [0.0 to 47.0]**	**41.0 [0.0 to 54.0]**	< 0.001

*Abbreviations*: *ARDS* Acute respiratory distress syndrome, *V*_*T*_ Tidal volume; *PBW* Predicted bodyweight, *IQR* Interquartile range, *N* Number, *PEEP* Positive end–expiratory pressure, *P*_*max*_ Maximum airway pressure, *ΔP* Driving pressure, *MP* Mechanical power, C_RS_ Respiratory system compliance, *FiO*_*2*_ Fraction of inspired oxygen, *RR* Respiratory rate, *PaO*_*2*_ Partial pressure of arterial oxygen, *PaCO*_*2*_ Partial pressure of arterial carbon dioxide, *VFD* Ventilator–free days and alive

Prone positioning and neuromuscular blocking agents were more often used in COVID–ARDS patients than in CLASSIC–ARDS patients (Table 2). COVID–ARDS patients received a tracheostomy more often than CLASSIC–ARDS patients.

**Fig. 3:**
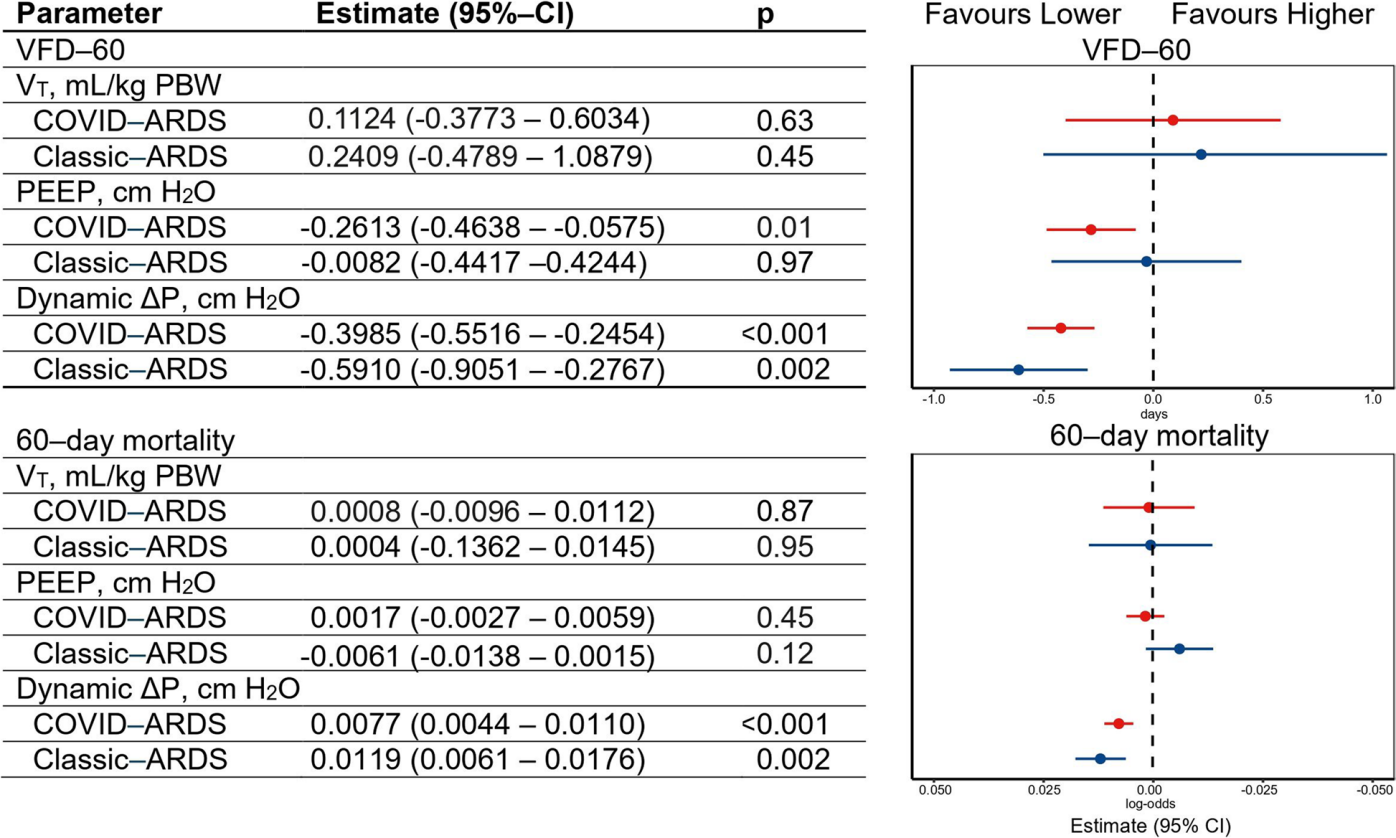
Mortality and ventilator–free days and Alive at day–60, and associations with ventilator parameters. The estimate is the average effect of the predictor variable on the response variable, while controlling for the other variables in the model. A positive estimate suggests a proportional effect, whereas a negative estimate suggests an inversely proportional effect. Abbreviations: ARDS = acute respiratory distress syndrome; VFD = ventilator–free days and alive; IQR = interquartile range; N = number; CI = confidence interval; V_T_ = tidal volume; PBW = predicted bodyweight; PEEP = positive end–expiratory pressure; ΔP = driving pressure

Mortality at day 60 was higher in COVID–ARDS patients compared to CLASSIC–ARDS patients (Table 2 and Fig. 3), and COVID–ARDS patients had significantly less VFD–60. Following multivariable adjustment, higher ΔP had an association with higher 60–day mortality and less VFD–60 in both groups. Higher PEEP also had an association with less VFD–60, but only in COVID–ARDS patients and not in CLASSIC–ARDS patients. In both groups, V_T_ neither had an association with 60–day mortality nor with VFD–60 (eFigure 3 and eFigure 4).

## Discussion

We pooled the individual data of patients from six observational studies of ventilation and compared ventilation characteristics and associations with outcomes between COVID–ARDS with CLASSIC–ARDS. The main findings were: (1) compared to CLASSIC–ARDS patients, COVID–ARDS patients were ventilated with lower V_T_ and higher PEEP, at lower ΔP and higher Crs, however with a higher MP; (2) 60–day mortality was not different between COVID–ARDS and CLASSIC–ARDS, but COVID–ARDS patients had less VFD–60; (3) higher ΔP had an association with higher 60–day mortality and less VFD–60 in COVID–ARDS and CLASSIC–ARDS; and (4) higher PEEP also had an association with less VFD–60, but only in COVID–ARDS.

Our findings add to the current understanding of differences and similarities between COVID–19 ARDS patients and pre–COVID ARDS patients. The international design of our study increases the generalizability of the findings across diverse healthcare systems, both in ARDS patients caused by COVID–19 and in patients with ARDS due to pneumonia from before the pandemic. The large sample size and high quality of the collected data allowed for sophisticated analyses of epidemiology, respiratory support strategies, and outcomes. Additionally, we found associations between key ventilator settings and patient outcomes.

Several studies have compared COVID–19 ARDS with pre–COVID ARDS. The epidemiological differences between COVID–19 ARDS and pre–COVID ARDS patients in our study align with previous findings [21]. As with other studies [22, 23], we also found significant differences in ventilator variables like V_T_, PEEP, and ΔP, and in the use of adjunctive therapies. Our study contributes by demonstrating these differences specifically among ARDS patients and comparing COVID–19 ARDS to pre–COVID ARDS due to respiratory infections. Differences in outcomes found in our study are, at least in part, in line with prior research findings [21, 23]. Our findings confirm that there are differences in mortality and the number of VFD–60 between COVID–19 ARDS and pre–COVID ARDS patients. However, these difference disappeared after propensity matching. This is important as it shows that, at least when comparing outcomes in ARDS patients from an infectious cause, outcomes are not different, opposite to what was thought at the start of the pandemic.

We observed more frequent use of lower V_T_ in COVID–ARDS compared to CLASSIC–ARDS. Indeed, proportions of COVID–ARDS patients that received ventilation with a V_T_ < 6 or between 6 and 8 ml/kg PBW was higher than in CLASSIC–ARDS patients. This finding can be explained in several ways––e.g., it could be that the use of lung–protective ventilation with a lower V_T_ has improved in the last decade [15]. It is also conceivable that, at least early in the pandemic care for COVID–ARDS patients was provided by inexperienced ICU staff which could have been more adherent to existing guidelines for management of patients with ARDS [10, 24]. It is also possible that use of low V_T_ in COVID–ARDS is easier to control––these patients were often deeply sedated and paralyzed allowing a stricter adherence to lower V_T_. Of note, especially in those patients, ventilation with a lower V_T_ might be more beneficial than in spontaneous breathing patients [25].

Higher PEEP was more often used in COVID–ARDS patients than in CLASSIC–ARDS patients, at any FiO_2_ level. Indeed, proportions of COVID–ARDS patients that received ventilation with a PEEP between 8 and 12 cmH_2_O and even between 12 and 16 cmH_2_O was higher than in CLASSIC–ARDS patients. This finding can also be explained in several ways––e.g., a preference for use of higher PEEP in COVID–ARDS patients may have been triggered by the severity of ARDS, as COVID–ARDS was more often classified as moderate or severe, and more severe hypoxaemia naturally triggers the use of higher PEEP if PEEP/FiO_2_ tables are used. It is also possible that higher PEEP was used in the assumption that lung lesions with COVID–ARDS are more recruitable than in CLASSIC–ARDS. This may at least explain the lower ΔP and higher Crs in COVID–ARDS patients.

In COVID–ARDS patients, mechanical power exceeded that of CLASSIC–ARDS, even though the driving pressure was lower. This observation marks the significance of considering factors beyond driving pressure, such as respiratory rate and PEEP, when evaluating the protective nature of invasive ventilation. These findings emphasize the complexity of respiratory management in COVID–ARDS and the need for a comprehensive approach to optimize lungprotective ventilation strategies.

COVID–ARDS patients received prone positioning more often than CLASSIC–ARDS patients. Before the pandemic, prone positioning remained underused, probably because it was more considered a rescue therapy for refractory hypoxaemia [26]. While we cannot rule out that use of prone positioning increased already before the pandemic, we favour the idea that the higher use of prone positioning in COVID–ARDS patients was triggered by the more severe hypoxaemia in COVID–ARDS patients.

Our analysis found several associations between ventilation parameters and outcome. The association of higher ΔP with higher 60–day mortality and less VFD–60 is in line with previous studies [27–29]. The association of higher PEEP with worse outcome confirms the findings of earlier studies [30, 31]. Of note, this association was only found for COVID–ARDS. This may have been caused by the more frequent use of higher PEEP in COVID–ARDS than in CLASSIC–ARDS. One reason for the association between higher PEEP and worse outcome may be that sicker patients, with a higher chance of dying and prolonged ventilation, received higher PEEP than patients that were less sick. Nonetheless, a high PEEP is suggested to have detrimental effects [32], emphasizing the need to determine the optimal PEEP level based on lung recruitability rather than hypoxemia alone. Actually, one analysis of PRoVENT–COVID suggested worse outcomes if patients received ventilation according to a higher PEEP/lower FiO_2_ table as compared to ventilation according to a lower PEEP/higher FiO_2_ [30]. A post–hoc Bayesian analysis of a randomised clinical study, named the ‘Alveolar Recruitment for ARDS Trial’ (ART), wherein patients were randomized to receive ventilation with PEEP titrated to the best Crs and aggressive recruitment manoeuvres versus ventilation with a low PEEP strategy, suggested that higher PEEP with recruitment manoeuvres worsens the outcome of ARDS from pneumonia, while it may be beneficial in ARDS from another cause [33]. A posthoc analysis of a randomised clinical study named ‘Lung Imaging for Ventilator Setting in ARDS trial’ (LIFE), suggest that higher PEEP worsens outcomes in patients with ARDS with lesions that may not be recruitable with higher PEEP [34].

The findings of this pooled analysis extend the existing knowledge of the epidemiology, management of invasive ventilation and outcomes in COVID–ARDS. Our study shows that lung–protective ventilation was applied well in COVID–ARDS, and was comparable to best practice used in management for patients with CLASSIC–ARDS. Additionally, the effect of PEEP on major outcomes may have implications for care. At least it should trigger new studies that directly compare different PEEP strategies. Meanwhile, it could be more attractive to not use higher PEEP by default.

Our study has several strengths. We managed to receive and merge the datasets of four large observational studies of ventilation conducted in the COVID–19 pandemic with two well–performed pre–pandemic observational studies of ventilation––these six studies all focused on ventilation management and reported outcomes of invasively ventilated ARDS patients, allowing a robust analysis of ventilation management and the impact of certain ventilation parameters on outcome. While the COVID–19 studies were all national investigations, they are from different regions worldwide and were conducted in different types of hospitals, which increases the generalizability of our findings. The datasets from the original studies were rich and comprehensive, encompassing baseline and demographic data, granular ventilator settings and ventilation variables, and key clinical outcomes. All data could be harmonized and merged into one database.

We had an analysis plan in place before cleaning and closing of the new database, and this plan was strictly followed. The large numbers of patients allowed us to perform sophisticated statistical analyses of associations with outcomes.

This study has limitations. First, individual data was obtained from observational studies, which limits the ability to establish causality. Additionally, the willingness of data sharing could have led to selection bias towards the inclusion of ICUs with an interest in invasive ventilation and management of ARDS in the original studies. Second, studies in COVID–ARDS were conducted early in the COVID–19 pandemic, during which inexperienced staff and resource limitations could have influenced clinical decision making. Third, data was collected early in the pandemic when patient care took priority over data collection, resulting in more missing data than in previous studies. This affects the completeness and may impact the accuracy of our analysis. Fourth, we only reported on ventilation characteristics on day 1 and 2, because not all studies collected ventilation data beyond this timepoint. Therefore we were not able to compare ventilation management beyond day 2. Nevertheless, previous studies have shown ventilation characteristics don’t significantly change in the first four days after initiation of invasive ventilation [10]. Fifth, it is imperative to acknowledge the temporal distance between comparator cohorts. For the pre–COVID ARDS group we used patients of which data was collected between seven to nine years before the pandemic. We cannot exclude temporal differences, for instance due to studies that showed the importance of limiting liberal use of oxygen, and reducing the intensity of ventilation, e.g., by targeting a low driving pressure or a low mechanical power of ventilation, as well as the importance of early use of prone positioning. Sixth, is the lack of detailed subgroup analyses, particularly in patients with chronic respiratory comorbidities such as COPD. Although recent findings from a post–hoc analysis of the PRoVENT–COVID study by Tripipitsiriwat et al. [35] indicated that ventilation parameters did not show significant differences between COPD and non–COPD patients, it could be interesting to explore these subgroups. However, it was beyond the scope of our primary endpoint. Conducting such detailed subgroup investigations would require careful consideration to ensure the data from all included studies are appropriate for this type of analysis.

Finally, all COVID–19 ARDS patients, by definition, had a viral pneumonia, while patients in the classic ARDS group had respiratory infections of which the pathogen was not collected. This is an important limitation, as ARDS from a viral respiratory infection may differ from ARDS due to bacterial pneumonia. Consistent with other studies comparing COVID–19 ARDS to ARDS caused by other viruses, we found that the duration of ventilation was longer, and mortality was higher [21, 36, 37].

## Conclusions

Epidemiology and key ventilation characteristics were different in patients with COVID–ARDS compared to CLASSIC–ARDS, also ΔP was lower in COVID––ARDS patients. ΔP had an independent association with outcome in both groups, whereas PEEP had an independent association with outcome only in COVID–ARDS patients.

## Supplementary Information

Supplementary Material 1

## Data Availability

Data sharing: A de–identified dataset can be made available upon request to the corresponding authors one year after publication of this study, but only after permission of the principal investigators of all original studies. The request must include a statistical analysis plan.
