# Correction to: Epidemiology, ventilation management and outcomes of COVID–19 ARDS patients versus patients with ARDS due to pneumonia in the Pre–COVID era

**DOI:** 10.1186/s12931-025-03176-y

**Published:** 2025-03-28

**Authors:** Fleur–Stefanie L. I. M. van der Ven, Siebe G. Blok, Luciano C. Azevedo, Giacomo Bellani, Michela Botta, Elisa Estenssoro, Eddy Fan, Juliana Carvalho Ferreira, John G. Laffey, Ignacio Martin–Loeches, Ana Motos, Tai Pham, Oscar Peñuelas, Antonio Pesenti, Luigi Pisani, Ary Serpa Neto, Marcus J. Schultz, Antoni Torres, Anissa M. Tsonas, Frederique Paulus, David M. P. van Meenen, for the ERICC–, LUNG SAFE–, PRoVENT–COVID–, EPICCoV–, CIBERESUCICOVID–, SATI–COVID–19–investigators, ERICC–, LUNG SAFE–, PRoVENT– COVID–, EPICCoV–, CIBERESUCICOVID–, SATI– COVID–

**Affiliations:** 1 Department of Intensive Care, Amsterdam University Medical Centers, Location ‘AMC’, Meibergdreef 9, 1105 AZ Amsterdam, The Netherlands; 2 Department of Intensive Care, Rode Kruis Ziekenhuis, Beverwijk, The Netherlands; 3 Department of Emergency Medicine, Hospital das Clinicas HCFMUSP, Faculdade de Medicina, Universidade de São Paulo, São Paulo, Brazil; 4 Department of Intensive Care, Hospital Israelita Albert Einstein, São Paulo, Brazil; 5 Centre for Medical Sciences (CISMed), University of Trento, Trento, Italy; 6 Department of Anesthesia and Intensive Care, Santa Chiara Hospital, APSS Trento, Trento, Italy; 7 Department of Intensive Care, Hospital Interzonal de Agudos General San Martin La Plata, Buenos Aires, Argentina; 8 Interdepartmental Division of Critical Care Medicine, University of Toronto, Toronto, ON Canada; 9 Department of Pulmonology, Instituto Do Coracao (InCor), Hospital das Clinicas HCFMUSP, Faculdade de Medicina, Universidade de Sao Paulo, São Paulo, Brazil; 10 Department of Intensive Care, AC Camargo Cancer Center, São Paulo, Brazil; 11 Brazilian Research in Intensive Care Network (BRICNet), São Paulo, Brazil; 12 Department of Anaesthesiology and Intensive Care, Galway University Hospital, Saolta Hospital Group, Galway, Ireland; 13 School of Medicine, University of Galway, Galway, Ireland; 14 Department of Intensive Care, Multidisciplinary Intensive Care Research Organization (MICRO), St James’ Hospital, Dublin, Ireland; 15 Department of Intensive Care, Hospital Clínic de Barcelona, Barcelona, Spain; 16 Departement of Pulmonology, Institut d’Investigacions Biomèdiques August Pi I Sunyer (IDIBAPS), Hospital Clínic de Barcelona, Barcelona, Spain; 17 Centro de Investigación Biomédica en Red en Enfermedades Respiratorias (CIBERES), Institute of Health Carlos III, Madrid, Spain; 18 Equipe d’Epidémiologie Respiratoire Integrative, Université Paris–Saclay, Paris, France; 19 Service de Médecine Intensive-Réanimation, DMU CORREVE, FHU SEPSIS, Groupe de Recherche Clinique CARMAS, Hôpital de Bicêtre, Paris, France; 20 Department of Intensive Care, Hospital Universitario de Getafe, Getafe, Spain; 21 Fondazione IRCCS Ca’ Granda Ospedale Maggiore Policlinico, Milan, Italy; 22 Department of Anesthesia and Intensive Care, Miulli Regional Hospital, Acquaviva Delle Fonti, Italy; 23 Australian and New Zealand Intensive Care Research Centre (ANZIC–RC), Monash University, Melbourne, Australia; 24 Mahidol–Oxford Tropical Medicine Research Unit (MORU), Mahidol University, Bangkok, Thailand; 25 Nuffield Department of Medicine, University of Oxford, Oxford, UK; 26 Department of Anesthesia, General Intensive Care and Pain Management, Division of Cardiothoracic and Vascular Anesthesia & Critical Care Medicine, Medical University of Vienna, Vienna, Austria; 27 Laboratory of Experimental Intensive Care & Anaesthesiology (L·E·I·C·A), Amsterdam UMC, Location AMC, Amsterdam, The Netherlands; 28 University of Barcelona, Barcelona, Spain; 29 Catalan Institution for Research and Advanced Studies (ICREA), Barcelona, Spain; 30 Center of Expertise Urban Vitality, Faculty of Health, Amsterdam University of Applied Sciences, Amsterdam, The Netherlands; 31 Department of Anaesthesiology, Amsterdam UMC, Location AMC, Amsterdam, The Netherlands

**Correction to: Respiratory Research (2024) 25:312**

10.1186/s12931-024-02910-2

In the original publication of this article [1], the collaborators “ERICC^a^–,LUNG SAFE^b^–, PRoVENT–COVID^c^–, EPICCoV^d^–, CIBERESUCICOVID^e^– and SATI–COVID–19^f^–investigators” from the original studies were not identified in HTML due to incorrect tagging.

The original article has been corrected.
